# Mixed convection in thermo-gravitational column: a continuous species separation

**DOI:** 10.1140/epje/s10189-025-00512-4

**Published:** 2025-08-05

**Authors:** Khairi Sioud, Abdelkader Mojtabi, Marie-Catherine Charrier-Mojtabi, Ali Abdennadher, Alain Bergeon

**Affiliations:** 1 Institut de Mécanique des Fluides de Toulouse, UMR CNRS INPT/UPS N 5502, Université de Toulouse, Toulouse, 31400 France; 2 Université de Carthage, EPT, LIM (LR 01-ES-13), Université de Carthage, La Marsa, 2078 Tunisie

## Abstract

So far, species separation has been achieved in closed vertical thermogravitational columns (TGC). To obtain continuous separation, the initially homogeneous binary solution with a positive thermodiffusion coefficient was introduced at a constant volumetric flow rate through one of the two vertical slots of the TGC and retrieved through the opposite slot. This process required the horizontal dimension separating the two slots to be sufficiently large for the mass regime at the exit slot to reach the steady state associated with a vertical stratification of the mass fraction. Analytical resolution and numerical simulations were developed and showed good agreement between theoretical and numerical results.

## Introduction

When an initially homogeneous binary mixture is subjected to a thermal gradient, mass transfer occurs, leading to heterogeneity of the mixture. The resulting change in the mass fraction of the mixture is known as thermodiffusion or the Soret effect. In addition to the usual expression of the mass flux density vector $\textbf{J}_m$ given by Fick’s law, a part due to the temperature gradient is added such that:

1 $$\begin{aligned} {\textbf {J}}_m =-\rho D \nabla C-\rho {D_T} {\nabla } T \end{aligned}$$

where $\rho$, *D* and $D_T$ are respectively, the density of the binary mixture, the Fick’s diffusion coefficient and the thermodiffusion coefficient of the densest component of mass fraction *C*, and *T* is the local temperature.

**Fig. 1:**
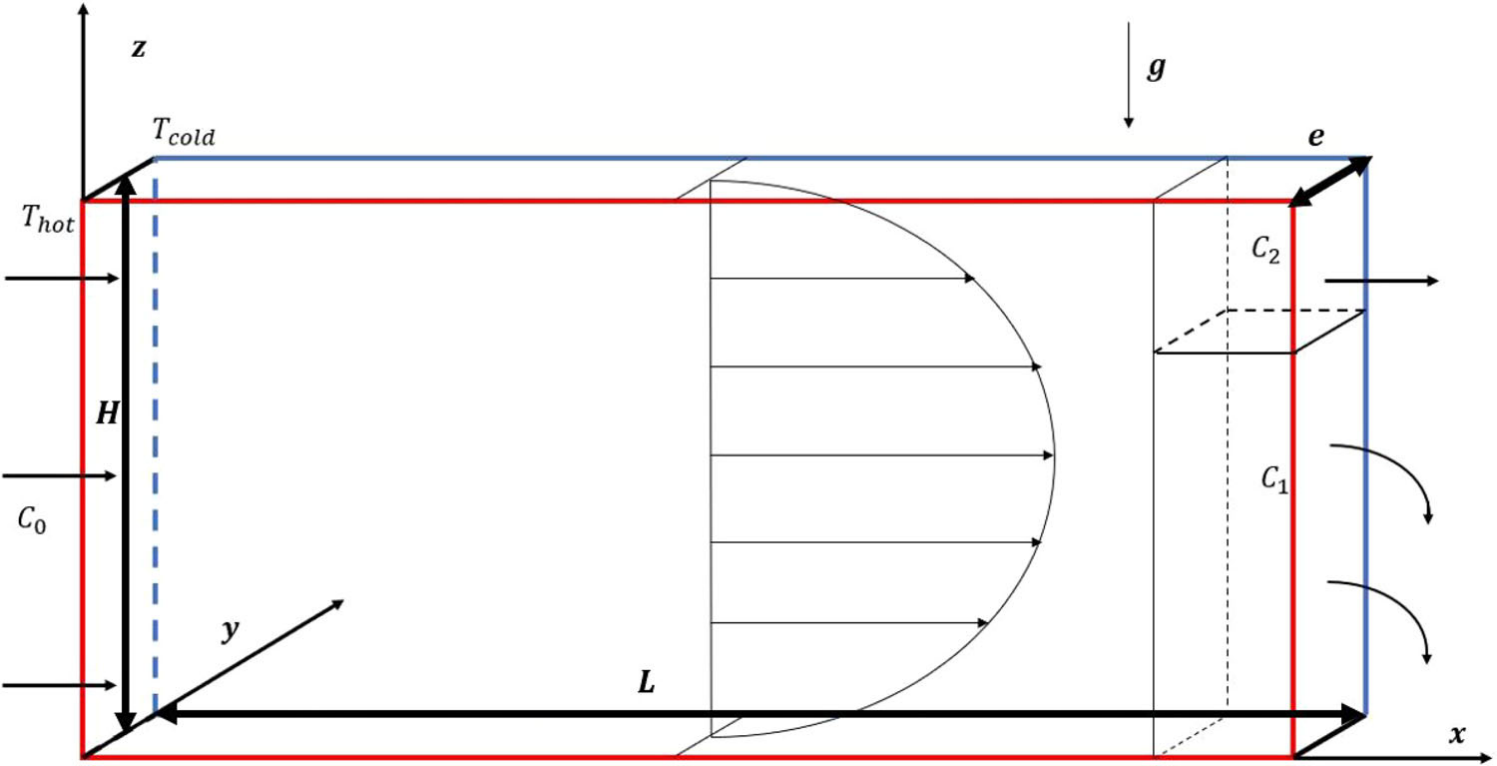
Sketch of the thermogravitational cell

Thermo-gravitational diffusion has been widely studied because of its many fundamental and industrial applications. [9, 14, 18] have summarized some of the multiple applications and Furry, [5] carried out a theoretical study of the Soret effect in 1939. These authors, in order to obtain an analytical solution to the problem of thermogravitational convection in a vertical column, used the so-called “forgotten effect” hypothesis. This involves not considering the mass fraction in the expression of density in the Navier Stokes equations. In 1959, [10] used thermogravitational columns (TGCs) consisting of a porous medium saturated by a binary mixture, which made it possible to work with cells of greater thickness and thus to obtain a greater amount of separated product. Over the years, [1, 6–8] and many other authors, notably [3] have carried out experimental work to measure thermodiffusion coefficients. Others have focused on increasing species separation by using different configurations, e.g. by studying vertical and inclined columns ([15] and [4]). In 2007, [2] carried out an analytical and numerical study of the stability of the flow due to the Soret effect in a horizontal porous cavity. In 2019, [12] studied thermogravitational diffusion using mixed convection and the Soret effect in a horizontal cavity with two opposing moving walls. In order to optimize the species separation, [13] changed the configuration by studying this phenomenon in annular horizontal porous cavities and, more recently, [17] compared species separation between the horizontal and vertical annular cylindrical configurations in terms of species separation values and the amount and time needed to reach steady states. More recently, [11] investigated linear stability of the conductive state of a ternary mixture of hydrocarbons in a horizontal porous layer. In the present work, we consider a new configuration combining forced convection with thermogravitational diffusion. The thermogravitation cell is parallelepipedic, vertical and differentially heated on the two vertical walls facing each other. The binary fluid enters at $x=0$ with a constant volumetric flow rate and a homogeneous composition, and comes out at $x=L$. Once mass regime is reached, it is possible to separate the binary fluid of mass fraction $C_1$ from the binary fluid of mass fraction $C_2$ with $C_2 < C_1$. This same process is then repeated by introducing a thin separation blade where the component to be enriched, for example the binary fluid of mass fraction $C_2$, is introduced into another open TGC. This process use several TGCs together until the mixture having a mass fraction compatible with industrial or laboratory applications is obtained.

**Table 1:** Thermo-physical properties of water-ethanol mixture at an ambient temperature of $T_0=25^\circ$C.

$\rho _0$	$C_0$	$\nu$	$\alpha$
910.085	0.5	$23.44\times 10^{-7}$	$8.55 \times 10^{-8}$
$$\beta _C$$	$$\beta _T$$	*D*	$$D_T $$
$24.5\times 10^{-3}$	$0.885\times 10^{-3}$	$0.362\times 10^{-3}$	$1.77\times 10^{-12}$

**Fig. 2:**
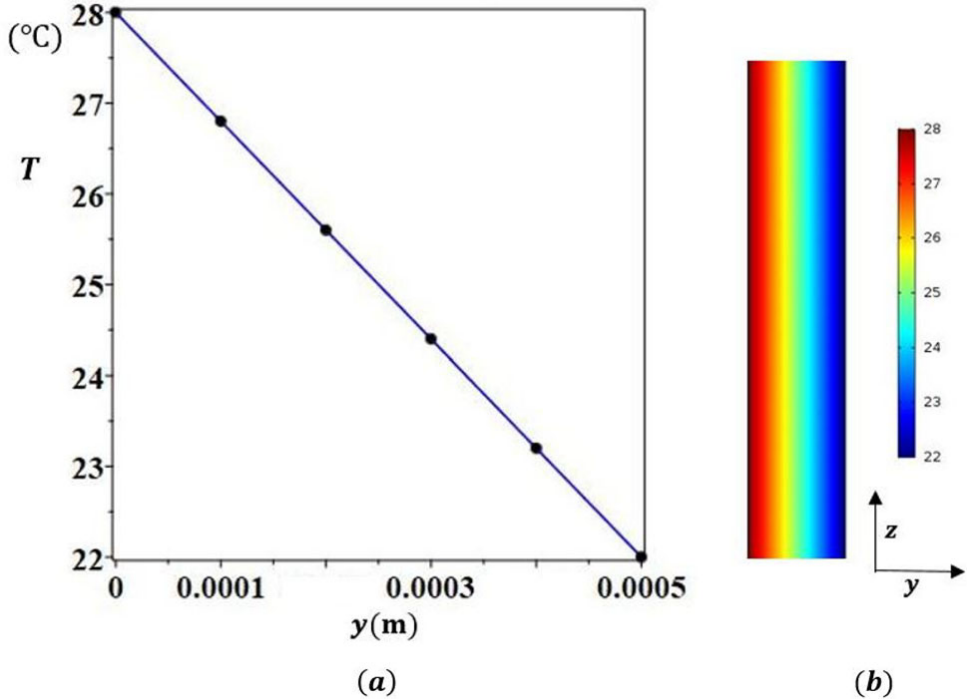
**(a)** Comparison between the analytical (continuous line) and the numerical (dots) results of temperature as a function of *y* **(b)** Numerical results of temperature field in the (*y*, *z*) plane

**Fig. 3:**
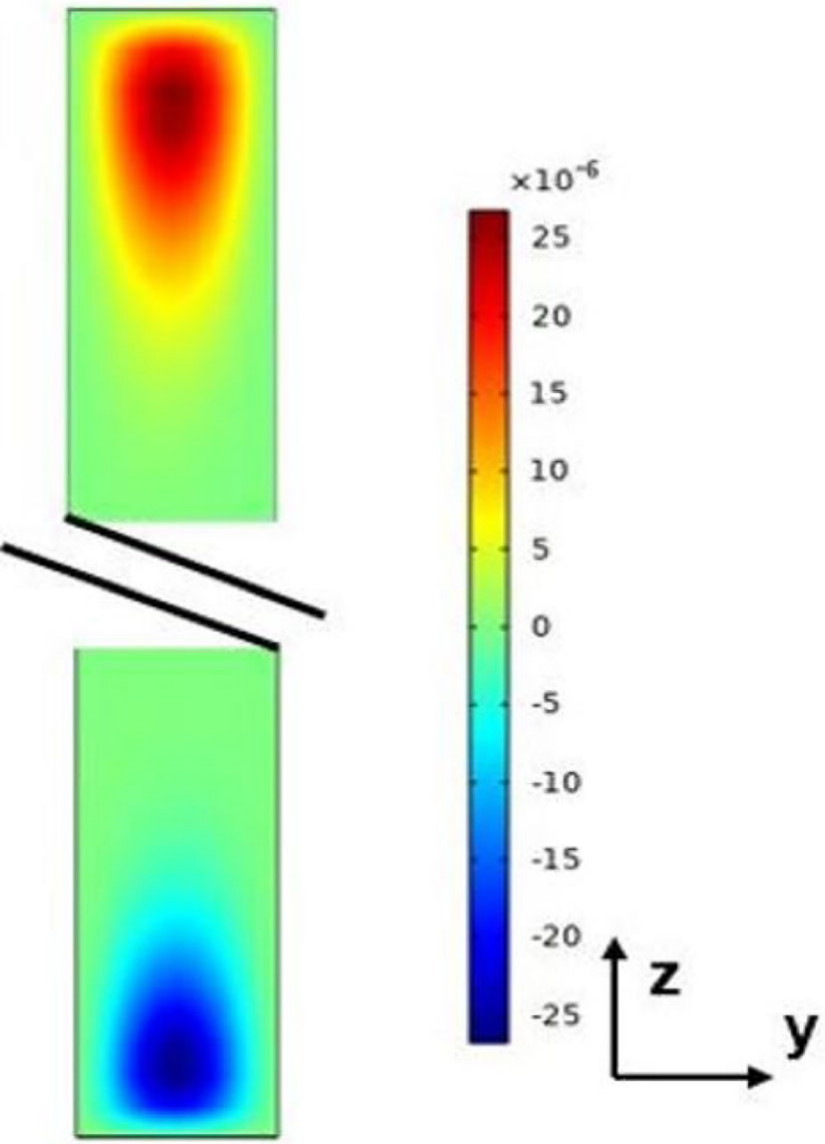
Numerical results of the velocity field in a plane $$\forall $$ $$ x= Cst$$

## Mathematical formulation

The vertical thermogravitational column considered opens at its faces $x=0$ and $x=L$, as shown in Figure 1. This parallelepipedic cell is of thickness *e* and height *H* with $e<<H<<L$. The two lateral vertical walls at $y=0$ and $y=e$ are maintained at different and constant temperatures $T_h$ and $T_c$, respectively, and the horizontal walls are perfectly insulated. The binary water-ethanol mixture (50%), initially homogeneous, enters through the slot $x=0$ with a constant volumetric flow rate and with a mass fraction $C_0=0.5$. A thin horizontal blade placed at the end of the TGC at $x=L$ and $z=z_1$, separates the initially homogeneous mixture into a mixture of mass fraction $C_1$ delimited by $z=0$ and $z=z_1$ and a mixture of mass fraction $C_2$ delimited by $z=z_1$ and $z=H$, with $C_2 < C_1$. The analytical and numerical results found were compared with an experimental result by considering a TGC of the same dimensions, $H = 30$ mm, $e = 0.5$ mm, as the one used in the experimental study by [16]. The thermophysical properties of the water-ethanol mixture of mass fraction $C_0=0.5$ are presented in Table 1 taken from [12], for an average temperature of $25^\circ$C. The thermogravitational column is open at $x=0$ and $x=L$, and the determination of the length *L* and the flow rate $U_d$ will be described later.

The governing equations of the problem are the continuity equation, the Navier-Stokes equation, and the energy and the species conservation Eq. (2):

2 $$\begin{aligned}  &   \nabla \cdot \textbf{V} = 0 \nonumber \\  &   \frac{\partial \textbf{V} }{\partial t}+(\textbf{V} \cdot \nabla ) \textbf{V}\nonumber \\    &   \quad =-\frac{\nabla P}{\rho _0}-(1-\beta _T(T-T_0)\nonumber \\    &   \qquad -\beta _C(C-C_0))g\textbf{z}+\nu \nabla ^2 \textbf{V} \nonumber \\  &   \frac{\partial {T}}{\partial {t}} +{\textbf{V}\cdot \nabla } T= \nabla \cdot (\alpha \nabla T) \nonumber \\  &   \frac{\partial {C}}{\partial {t}}+ {\textbf{V}\cdot \nabla }C= \nabla \cdot (D \nabla C +D_TC_0(1-C_0)\nabla T) \nonumber \\ \end{aligned}$$

The fluid is assumed to be incompressible and to obey the Boussinesq approximation. The viscous dissipations and are considered negligible. It is also assumed that the dynamic and thermal regimes are established at the inlet of the cell and that the length of the cell is sufficient for the mass fraction field at the outlet of the cell to be established. The boundary conditions of the problem are written as follows: For velocity ($\textbf{V}$):

3 $$\begin{aligned} x=0 ; ~~u=36 u_{deb} \frac{z}{H}\left( \frac{z}{H}-1\right) \frac{y}{e}\left( \frac{y}{e}-1\right) \nonumber \\ x=L; ~~\frac{\partial \textbf{V}}{\partial x}=0 \nonumber \\ y=0,e; ~~\textbf{V}=\textbf{0} \nonumber \\ z=0,H;~~ \textbf{V}=\textbf{0} \end{aligned}$$

**Fig. 4:**
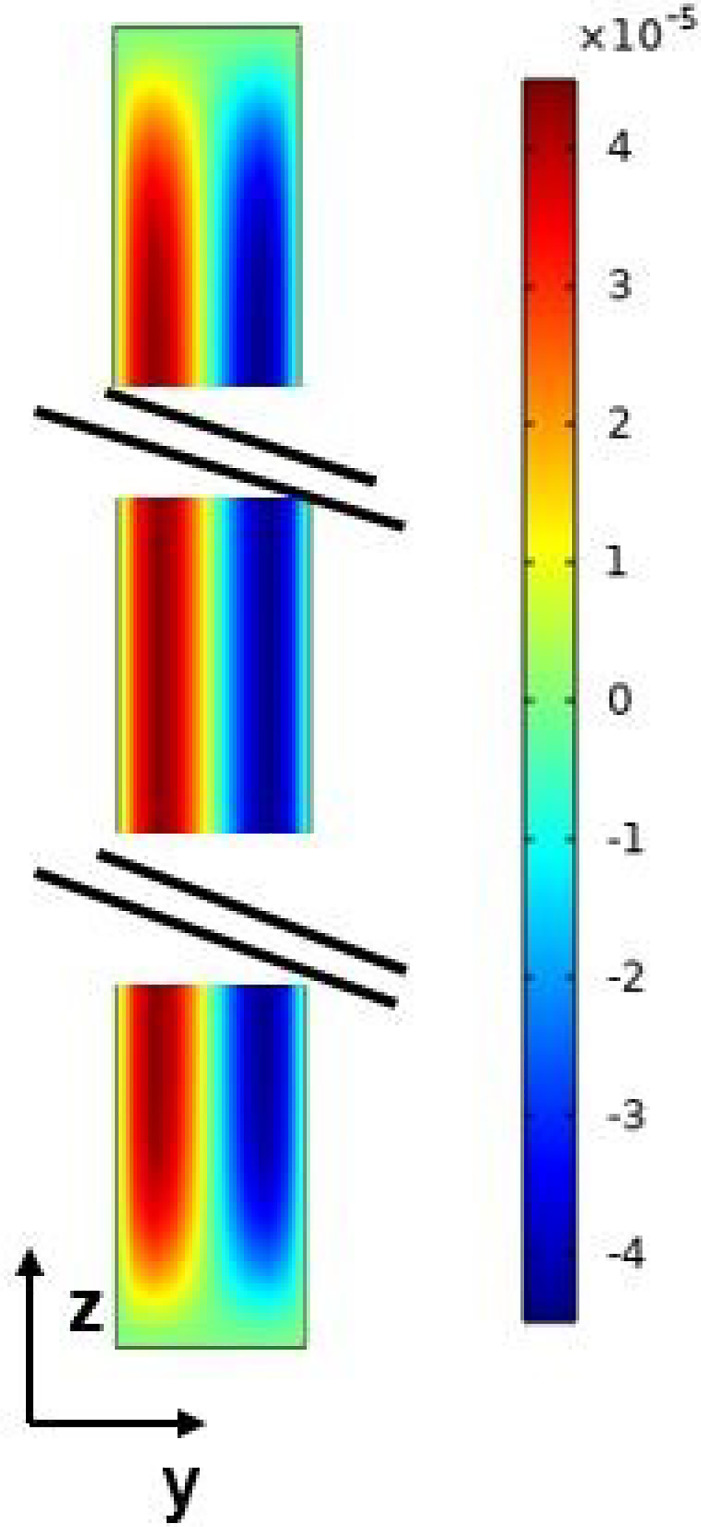
Numerical results of vertical velocity fields along $\textbf{z}$ for $x=\frac{L}{2}$

**Fig. 5:**
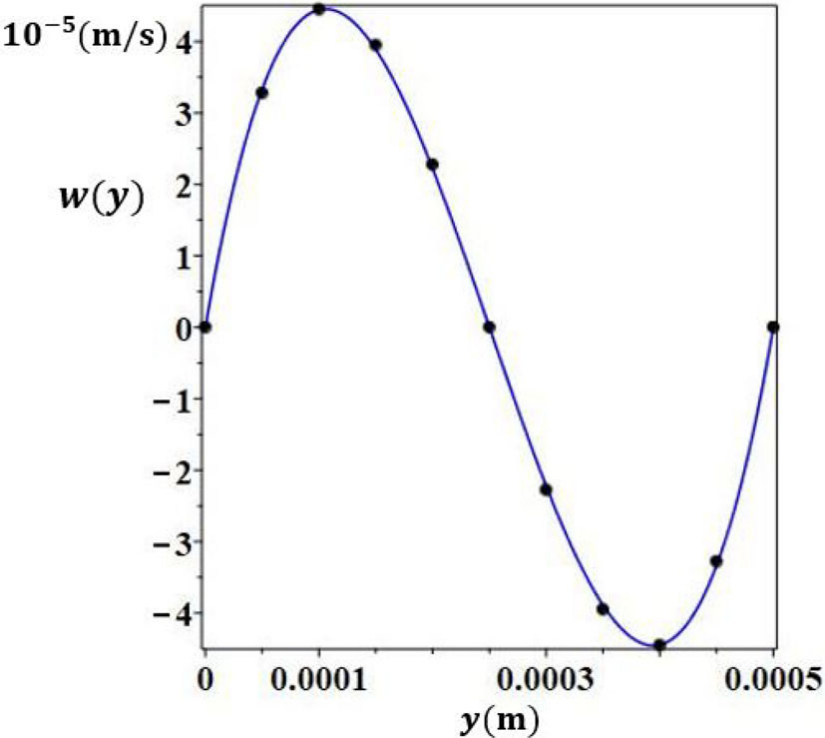
Comparison between the analytical (continuous line) and the numerical (dots) results of vertical velocity as a function of *y* at $z=\frac{H}{2}$ and $x=\frac{L}{2}$

**Fig. 6:**
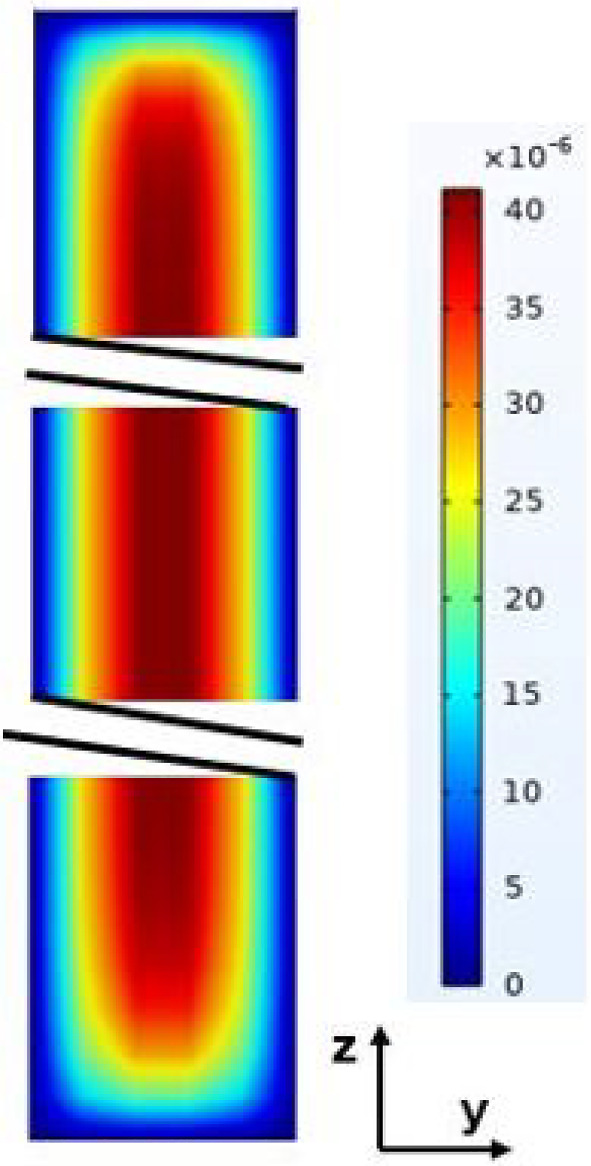
Numerical results of axial velocity fields at $x=\frac{L}{2}$

**Fig. 7:**
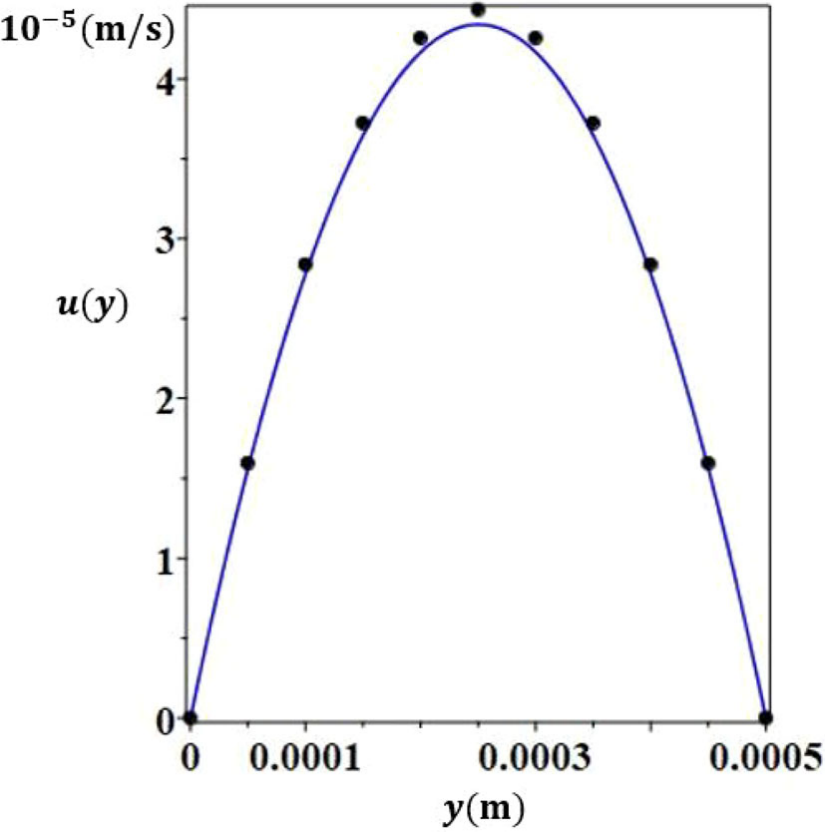
Comparison between the analytical expression (continuous line) and the numerical (dots) results of axial velocity as a function of *y*

**Fig. 8:**
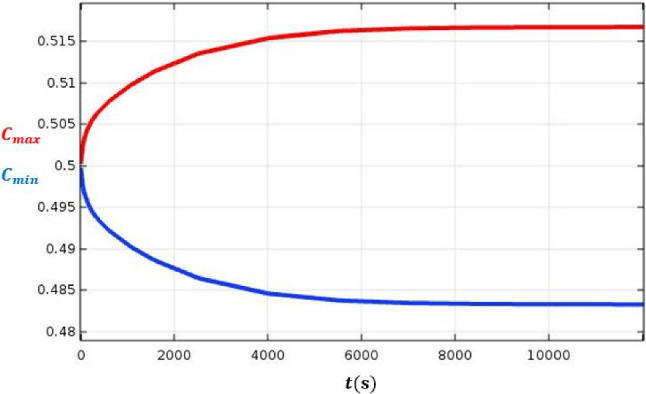
The evolution of maximum and minimum mass fraction versus time, this result is identical to that obtained in the TGC

For temperature (*T*):

4 $$\begin{aligned} x=0 ; ~~T=\frac{(T_f-T_c)y}{e} +T_c \nonumber \\ y=0; ~~ T=T_c \nonumber \\ y=e; ~~ T=T_f \nonumber \\ z=0,H; ~~\frac{\partial {T}}{\partial z}=0 \end{aligned}$$

For mass fraction (*C*):

5 $$\begin{aligned} x=0 ; ~~C=C_0 \nonumber \\ x=L; ~~\frac{\partial C}{\partial x}=0 \nonumber \\ y=0,e; ~~D\frac{\partial C}{\partial y}+D_TC_0(1-C_0)\frac{\partial T}{\partial y}=0 \nonumber \\ z=0,H; ~~\frac{\partial C}{\partial z}=0 \end{aligned}$$

## Analytical solution

To determine the steady-state analytical solution of the problem, it is assumed that, when the flow is established, all variables (*T*, $\textbf{V}$, *C*), in any cross-section perpendicular to the heated plates $x=cst$, are independent of *x*. We assume that the parallel flow approximation is valid in any $x=cst$ plane and we seek a solution of the form:

6 $$\begin{aligned} \textbf{V}= &   u(y,z)\textbf{x}+w(y)\textbf{z} \nonumber \\ T= &   h(y) \nonumber \\ C= &   mz+g(y) \end{aligned}$$

**Fig. 9:**
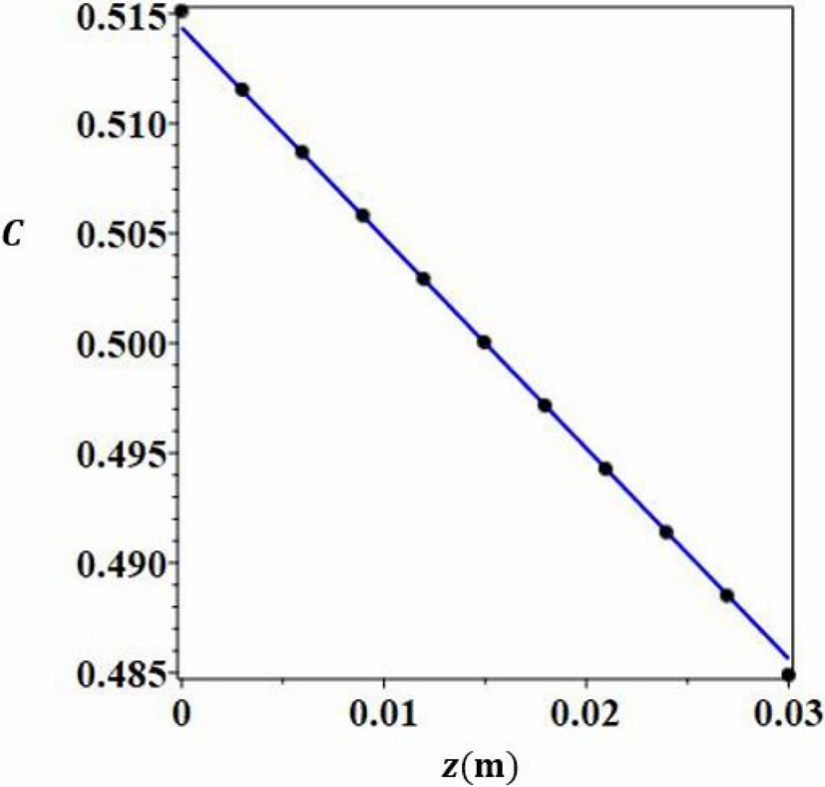
Numerical (dots) and analytical (continuous line) mass fraction values as a function of *z* for constant values of $y=e/2$ and $x=x_{lim}$

**Fig. 10:**
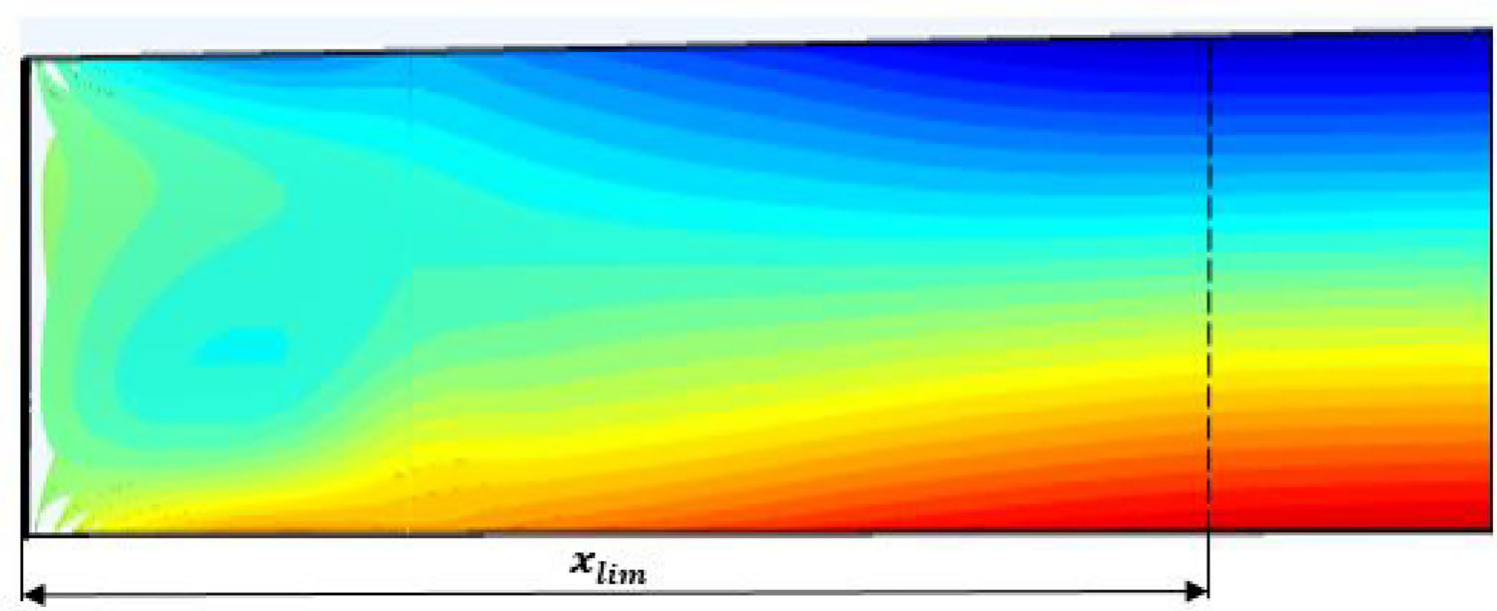
Numerical result of mass fraction field along *x*

Where *m* designates the mass fraction gradient along $\textbf{z}$. The expression of the component *u*(*y*, *z*) of the velocity $\textbf{V}$ along $\textbf{x}$ is given by the classical relation with the velocity is induced by forced convection alone. Thus, the simplified equations allowing the analytical solution to be determined are given by:

7 $$\begin{aligned}  &   \nu \frac{\partial ^3 w}{\partial y^3}+g(\beta _T\frac{\partial T}{\partial y}+\beta _C\frac{\partial C}{\partial y})=0 \nonumber \\  &   \frac{\partial ^2 T}{\partial y^2}=0 \nonumber \\  &   mw-D\frac{\partial ^2 C}{\partial y^2}-D_TC_0(1-C_0)\frac{\partial ^2 T}{\partial y^2}=0 \end{aligned}$$

By taking the boundary conditions and the conservation of volumetric flow rate and the mass flux through any cross-section $x=cst$ into account, we obtain system (8) below.

8 $$\begin{aligned}  &   w(y)=-\frac{g\beta _T\delta T}{12 \nu e}y(y-e)(2y-e) \nonumber \\  &   h(y)=\frac{T_f-T_c}{e}y+T_c \nonumber \\  &   g(y)=-\frac{\delta T}{1440 e\nu D}((e-y)G(y)\nonumber \\    &   \quad +720C_0(1-C_0)\nu D_T-720 e \nu (2C_0-Hm)) \nonumber \\  &   G(y)=\beta _Tgm(6y^4-12ey^3+4e^2y^2+2e^3y+e) \nonumber \\  &   m=\frac{504\nu g \beta _T\delta T^2e^2C_0(1-C_0)D_T}{g^2\beta _T^2\delta T^2 e^6-362880D^2\nu ^2} \end{aligned}$$

**Fig. 11:**
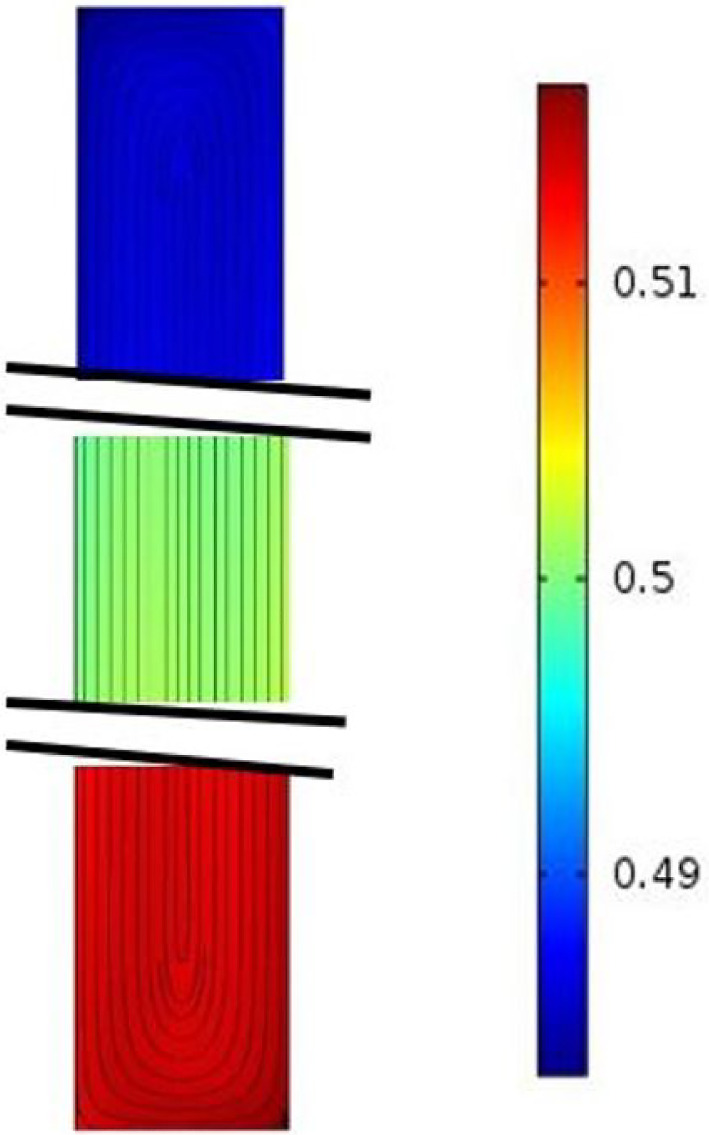
Stream function and mass fraction field in a plane $x=cst$ for $x \ge x_{lim}$

**Fig. 12:**
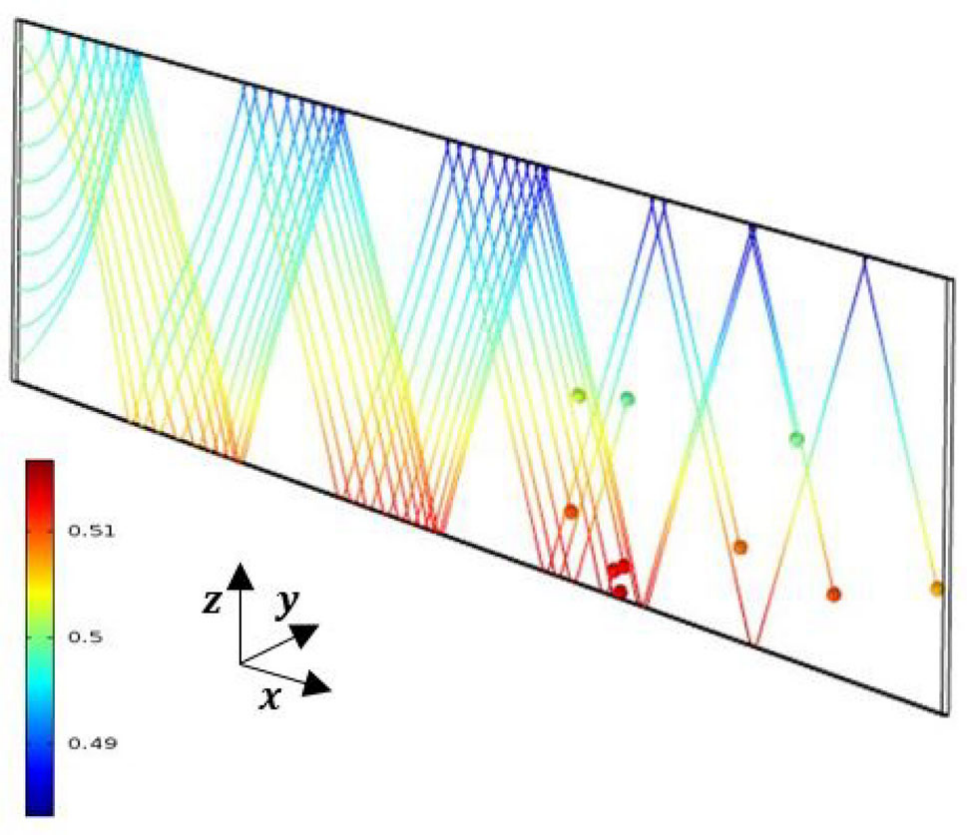
3D Numerical streak-lines, showing the mass fraction of fluid particles at $t=11 000$ s

## Results and discussion

### Comparison between analytical and numerical results

The analytical results obtained from solving (6) with the associated boundary conditions are compared to the results of direct numerical simulations, Eq. (2), and boundary conditions (3-5). The numerical simulations were carried out by a finite elements method using COMSOL Multiphysics software in 2D and 3D. The binary mixture considered, as mentioned in Table 1 was a $50\%$ water- $50\%$ ethanol mixture. Figure 2 reports the variation of *T* as a function of *y*: the analytical results (continuous line) and the numerical results (black dots) are in good agreement. It was checked that the velocity component along *y* was indeed null, so the validity of the parallel flow hypothesis for the determination of the analytical solution in the cross section plane (*y*, *z*) was well confirmed ($e<< H$), except near the ends of the cell (Fig. 3).

Figures 4 and 5 present the vertical component of the velocity *w*(*y*) in any plane $x=cst$, once the flow is established. The analytical results are in very good agreement with the numerical ones. Regarding axial velocity, $u_{deb}$ was chosen as follows in order to guarantee the validity of the analytical solution :

9 $$\begin{aligned} u_{deb}=\frac{\int _ {0}^ {e} |w(y)| dy}{e} \end{aligned}$$

**Fig. 13:**
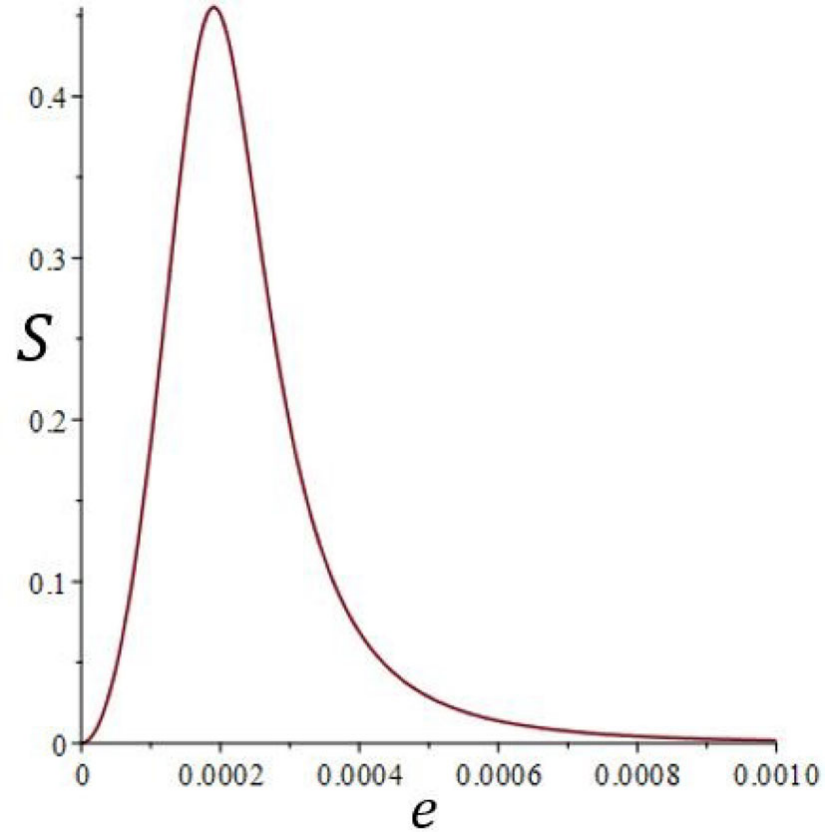
Separation, *S*, according to the thickness, *e*, for $H = 30$ mm

**Fig. 14:**
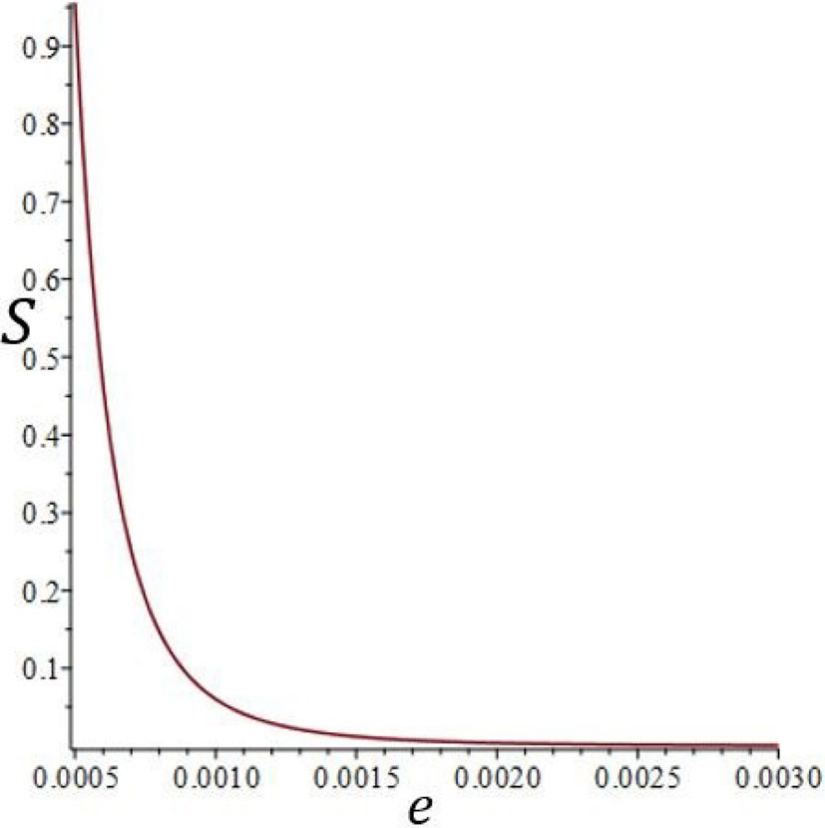
Separation, *S*, against thickness, *e* (m), for $H = 1$ m

**Fig. 15:**
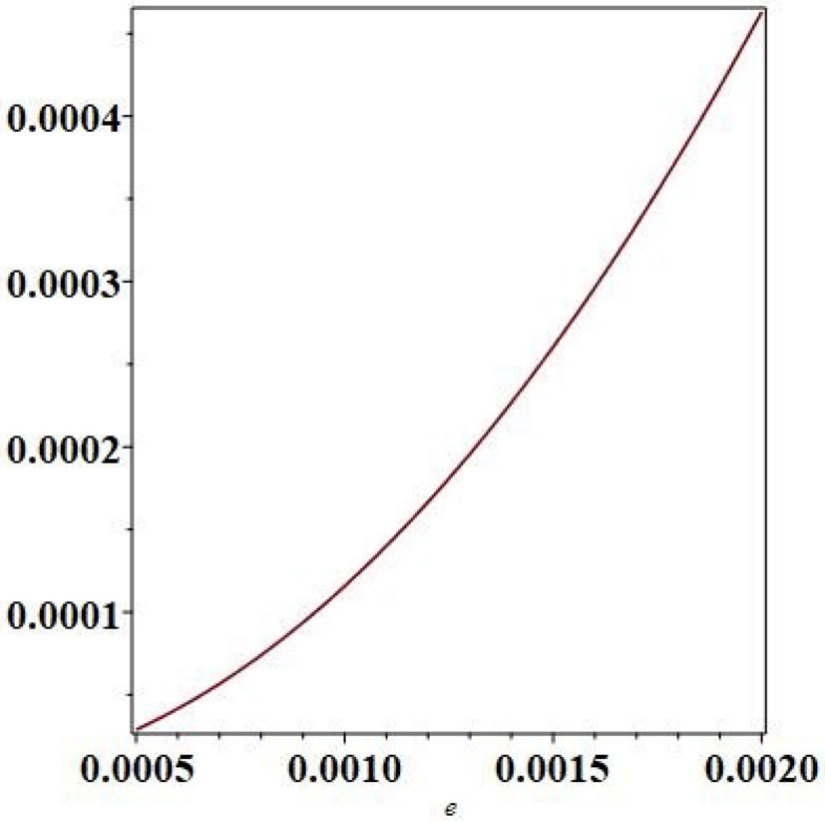
$$u_{deb}$$ against *e* for $$H=1$$m

**Fig. 16:**
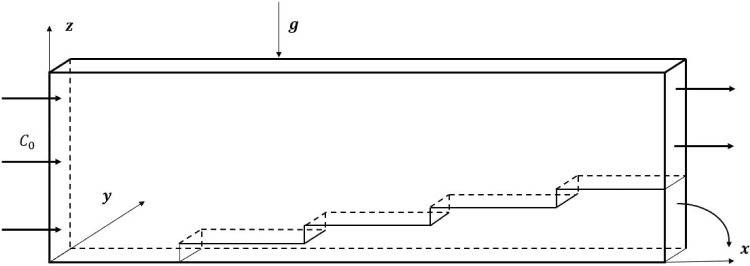
Schematic system (1) for obtaining continuous separation

Figures 6 and 7 show the good agreement between the results of the direct numerical simulations and the analytical ones for the axial velocity. These results lead to an accurate determination of $x=x_{lim}$, abscissa where the mass regime is fully established. The time needed to reach the stationary mass fraction state can be calculated from the 2D simulations as seen in Fig. 8. The mass fraction field evolves from the homogeneous initial state, $C_0$, to the stratified steady state in a time of about 11000 s.

**Fig. 17:**
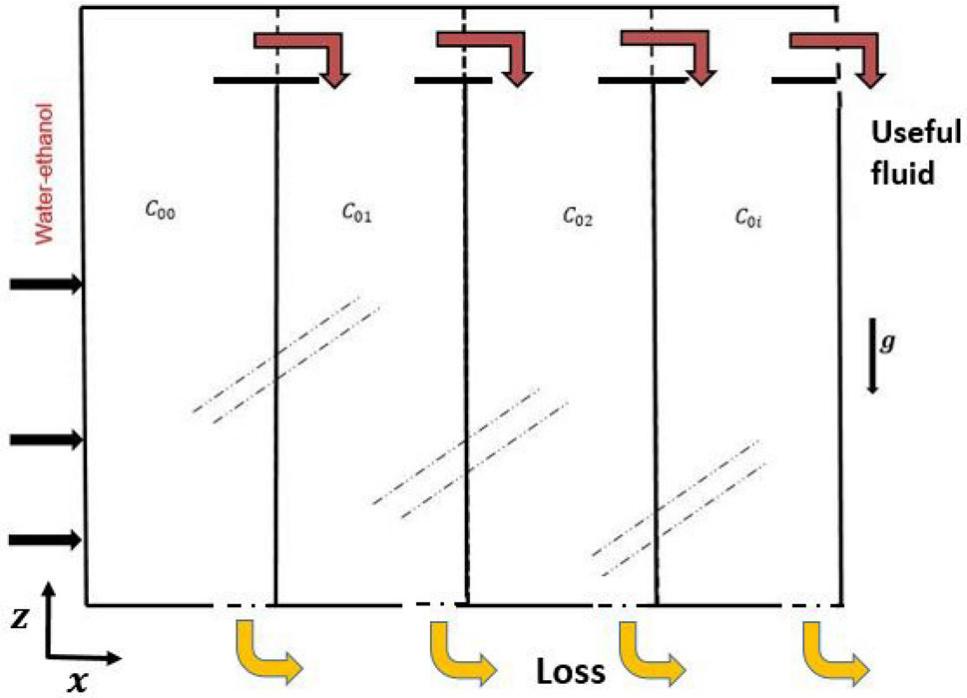
Schematic of system (2) to improve species separation

Figure 9 shows the very good agreement between analytical and numerical mass fraction values according to *z* for $y=e/2$ and $x=x_{lim}$. In Fig. 10, the evolution of the mass fraction field is presented as a function of *x* and *z* for $y=e/2$. It can be deduced that $x=x_{lim}= u_{deb} \times$(time to reach stationary sate for mass fraction field)$= 450$ mm for the parameters of this study. It can be seen from Figs. 8 and 10 that the stationary state of the mass regime is reached slightly more in the lower part of the column, $C_{max}$ than in the upper $C_{min}$.

The streamlines and the mass fraction field are presented in Fig. 11 and are invariant at each cross section $x \ge x_{lim}$. In Fig. 12, 3D numerical simulations allow streak-lines to be drawn, together with the mass fraction of several fluid particles taken at time $t=0$, at $x=0$, in the plane $y=e/2$ and for several values of *z* at a given time $t=11000$s. We also note in Fig. 12 the evolution of the mass fraction of these different particles during their evolution between the times $t=0$ and $t=11000$ s.

The separation, $S=C_{max}-C_{min}$, is plotted against *e* in Fig. 13 for $H=30$ mm. We note that the parameters used in this part of the study do not enable a relatively large amount to be achieved since $S\approx 0.03$. We will consider a cell of 1 m height for larger scale uses.

For practical uses of the species separation process, let us consider a cell 1 m high for a larger scale use: $H=1$ m, $\delta T=10^\circ$ C. Figure 14 shows a plot of the separation against the thickness *e* for $H=1$ m and $\delta T=10^\circ$ C. It can be seen that the separation becomes very significant when the thickness, *e*, approaches 0.5 mm. As the volumetric flow rate decreases when the thickness decreases as shown in Fig. 15, a compromise must be found between the separation value and the volumetric flow rate. We chose to continue the study with a thickness of $e=1$ mm.

### Continuous separation with serial cells

To increase the species separation in a continuous way, several thermogravitational columns were connected in series. The binary fluid considered was a mixture of water with 50%-ethanol [17]. Figure 16 presents the diagram of 5 TGC of the same thickness, $e=1$ mm, connected in series. The first column, of height $H_1=1$ m, has a length $x_{lim1}=4.5$ m. The 5 TGC are in decreasing order of height so that the binary fluid that comes out of the last column, of smallest height $H_5=0.6$ m ($H_5=H_1-0.1 \times 4$ m), contains only the binary fluid having the desired mass fraction. Table 2 shows that the species separation (of the lighter component ) is improved each time the binary fluid leaves column Hi to enter column $H_{i+1}$, with *i* ranging from 1 to $N>5$.

## Conclusion

This paper presents a new technique for combining forced convection with thermo-gravitational diffusion to obtain a continuous species separation process that would be of interest in both the academic and industrial fields. The governing equations and the associated boundary conditions have been solved numerically in 2D, with a finite element method using the Comsol Multiphysics software, and analytically, by taking the suitable approximations mentioned (parallel flow approximation since $e<<H$) into account. Analytical and numerical results are in good agreement with each other and also agree with the experimental results of the previous study [16] obtained for a TGC height of $H=30$ mm and thickness, *e*, of 0.5 mm (See Fig. 17). Then the numerical simulations were extended in 3D, for larger columns, $H=1$ m and $e=1$ mm. These numerical results were also developed in order to obtain a multi-stage separation system. By applying a suitable temperature gradient to a multi-stage separation system and with well-chosen geometrical parameters for the cells and thermo-physical parameters of the binary mixture, we not only achieved continuous species separation, but also obtained the required amount of species of interest.

## Data Availability

The Data used in this study were generated and analyzed using symbolic computation software (MAPLE 2024) and the finite element software (COMSOL Multiphysics). The results of this research are based on the PhD thesis work of Khairi SIOUD (http://thesesups.ups-tlse.fr/5526/1/2022TOU30251.pdf). The specific data used to validate our findings are available upon request from the authors. For any inquiries regarding the data , please contact the corresponding author : Mojtabi@imft.fr

## References

[1] M.M. Bou-Ali, O. Ecenarro, J. Madariaga, C.M. Santamaría, J.J. Valencia, Stability of convection in a vertical binary fluid layer with an adverse density gradient. Phys. Rev. **59**, 1250 (1999) 10.1103/physreve.62.1420 11088604

[2] M. Charrier-Mojtabi, B. Elhajjar, A. Mojtabi, Analytical and numerical stability analysis of soret-driven convection in a horizontal porous layer. Phys. Fluids **19**, 124104 (2007) 10.1140/epje/i2017-11527-3 28367593

[3] J. Dutrieux, J. Platten, G. Chavpeyer, M. Mounir Bou-Ali, On the measurement of positive soret coefficient. J. Phys. Chem. B **106**, 6104–6114 (2002)

[4] B. Elhajjar, A. Mojtabi, P. Costes que, M.-C. Charrier-Mojtabi, Separation in an inclined porous thermogravitational cell. nt J Heat Mass Transf 53:4844–4851 (2010)

[5] W. Furry, R. Jones, L. Onsager, On the theory of isotope separation by thermal diffusion. Phys. Rev. **55**(1083), 1095 (1939)

[6] P. Kolodner, H. Williams, C. Moe, Optical measurement of the soret coefficient of ethanol/water solutions. J. Chem. Phys. **88**, 6512 (1988)

[7] A. Koniger, B. Meier, W. Kohler, Measurement of the soret, diffusion, and thermal diffusion coefficients of three binary organic benchmark mixtures and of ethanol/water mixtures using a beam deflection technique. Philos. Mag. **89**, 907 (2010)

[8] E. Lapeira, M.M. Bou-Ali, J. Madariaga, C.M. Santamaria Thermodiffusion coefficients of water/ethanol mixtures for low water mass fractions. Microgravity Sci. Technol. **28**, 533 (2016)

[9] J. Legros, Y. Gaponenko, A. Mialdun, T. Triller, A. Hammon, C. Bauer, W. Köhler, V. Shevtsova Investigation of fickian diffusion in the ternary mixtures of water ethanol triethylene glycol and its binary pairs. Phys. Chem. Chem. Phys. **17**, 27713–27725 (2015) 10.1039/c5cp04745e 26434813

[10] M. Lorenz, A.H. Emery, The packed thermal diffusion column. Chem Engng Sci **11**, 6–23 (1959)

[11] T. Lyubimova, I. Shubenkov, oret-induced convection of ternary fluid in a horizontal porous layer heated from below. Phys of Fluids **35**(8), 084114 (2023)

[12] A. Mojtabi, A. Khouzam, Y. Loujaine, M.C. Charrier-Mojtabi, Analytical and numerical study of soret mixed convection in two sided lid-driven horizontal cavity: Optimal species separation. Int J of Heat and Mass transfer **139**, 1037–1046 (2019)

[13] A. Mojtabi, K. Sioud, A. Bergeon, M.C. Charrier-Mojtabi, numerical and analytical studies of soret-driven convection flow inside an annular horizontal porous cavity. Fluids MDPI **6**, 357 (2021)

[14] D. Nield, A. Bejan, Convection in Porous Media (1998)

[15] J. Platten, M.M. Bou-Ali, J. Dutrieux, Enhancement molecular separation in inclined thermogravitational columns. J. Phys. Chem. B **107**, 11763–11767 (2003)

[16] B. Seta, E. Lapeira, D. Dubert, F. Gavaldà, M.M. Bou-Ali, X. Ruiz, Separation under thermogravitational effects in binary mixtures. Eur. Phys. J. E **42**, 58 (2019) 10.1140/epje/i2019-11818-7 31089829

[17] K. Sioud, A. Abdennadher, A. Bergeon, S. Kaddeche, M.C. Charrier-Mojtabi, A. Mojtabi, Thermogravitational separation in porous vertical and horizontal cylindrical annular cells saturated by a binary mixture. Eur. Phys. J. E **45**, 45 (2022) 10.1140/epje/s10189-022-00204-3 35552892

[18] K. Vafai, *Handbook of Porous Media* (Boston, 2015)

